# A retrospective analysis of two tertiary care dizziness clinics: a multidisciplinary chronic dizziness clinic and an acute dizziness clinic

**DOI:** 10.1186/s40463-019-0336-9

**Published:** 2019-03-11

**Authors:** Phillip Staibano, Daniel Lelli, Darren Tse

**Affiliations:** 1 Faculty of Medicine, University of Ottawa, Ottawa, Ontario Canada; 2 Division of Neurology, Department of Internal Medicine, University of Ottawa, Ottawa, Ontario Canada; 3 Department of Otolaryngology—Head & Neck Surgery, University of Ottawa, The Ottawa Hospital, Civic Campus, 1053 Carling Avenue, Ottawa, ON K1Y 4E9 Canada

**Keywords:** Acute dizziness, Chronic dizziness, Vertigo, Retrospective, Multidisciplinary

## Abstract

**Background:**

Vertigo remains a diagnostic challenge for primary care, emergency, and specialist physicians. Multidisciplinary clinics are increasingly being employed to diagnose and manage patients with dizziness. We describe, for the first time in Canada, the clinical characteristics of patients presenting with chronic and acute dizziness to both a multidisciplinary chronic dizziness clinic (MDC) and a rapid access dizziness (RAD) clinic at The Ottawa Hospital (TOH).

**Methods:**

We performed a retrospective review of all patients presenting to the MDC and RAD clinics at TOH from July 2015 to August 2017.

**Results:**

Overall, 211 patients (median age: 61 years old) presented to the RAD clinic and 292 patients (median age: 55 years old) presented to the MDC. In the RAD clinic, 63% of patients had peripheral dizziness, of which 55% had BPPV, and only one patient had functional dizziness. Interestingly, only 25% of RAD diagnoses were concordant with emergency department diagnoses; moreover, only 33% of RAD patients had HiNTS completed, while 44% had CT scans, of which only one scan had an abnormal finding. Prior to assessment, all patients in the MDC had an unclear cause of dizziness. 28% of patients had vestibular dizziness and 21% had functional dizziness, of which 43% had persistent postural perceptual dizziness. Moreover, 12% of patients with functional dizziness also suffered from comorbid severe anxiety and depression.

**Conclusions:**

Dizziness is a heterogeneous disorder that necessitates multidisciplinary care, and clinics targeting both the acute and chronic setting can improve diagnostic accuracy, ensure appropriate diagnostic testing, and facilitate effective care plans for patients with dizziness.

## Introduction

Vertigo, dizziness, and unsteadiness are common symptoms in the general population [1] and account for up to 10% of patient complaints in emergency settings [2]. Lifetime prevalence of dizziness and vertigo have been estimated to be from 20 to 30% [3]. Dizziness can be caused by an array of etiologies, including central and peripheral vestibular disorders, central nervous system disease, psychiatric disease and cardiovascular processes [4]. Often, despite a thorough history and physical examination, diagnosing the cause or causes of dizziness remains a challenge for primary care physicians, emergency physicians, and specialists alike [5]. In addition, diagnostic tests are often unable to provide useful diagnostic information to further distinguish between causes of dizziness [6]. Over the past few decades, multidisciplinary dizziness clinics, which incorporate the clinical opinions of neurologists, otolaryngologists, and other specialists, have been established to manage patients with dizziness [7, 8].

The Multidisciplinary Dizziness Clinic (MDC) and the Rapid Access Dizziness (RAD) clinic are conducted out of The Ottawa Hospital (TOH), a high-volume academic teaching hospital in Ottawa, Canada. Both clinics provide simultaneous opinions from a neurologist and an otolaryngologist, in addition to gathering input from the clinic nursing team, audiologists, physiotherapists, and medical trainees. The MDC accepts referrals only from other specialists (i.e. neurology, otolaryngology, and internal medicine) with unclear causes of dizziness and as such, sees a homogenous focused subset of the most chronically dizzy patients. Following diagnosis and formulation of a treatment plan in the MDC, patients are discharged back to the care of the referring physician for active management of their dizziness. In contrast, the RAD clinic accepts referrals only from the TOH Emergency Department for patients presenting with acute dizziness of a maximum duration of four weeks, with no previous history of dizziness. Hence, the RAD clinic assesses a vastly different patient population than the MDC. The aim of the RAD clinic is to help patients achieve a full recovery when possible, or to anticipate and prevent the transformation of acute dizziness into chronic dizziness in susceptible populations. When combined, however, the MDC and RAD clinic provide a unique opportunity to treat and study two distinct patient populations that suffer from dizziness of varying duration and severity.

The objective of this retrospective study is to provide a comparison of the demographic, clinical, and diagnostic characteristics of patients who presented to two distinct tertiary dizziness clinics in a Canadian Academic Teaching Hospital setting.

## Multidisciplinary Dizziness Clinic & Rapid Access Dizziness Clinic Format

The MDC is staffed by the senior authors (DT and DL) and encompasses otolaryngology/neuro-otology, neurology/neuro-ophthalmology, audiology and nursing. All patients are assessed and managed by an allied health team. In addition, there is a local network of trained vestibular physiotherapists who support the MDC on a case-by-case basis. The MDC accepts referrals for undiagnosed dizziness of duration over 3 months. Referrals are accepted if made by a neurologist, otolaryngologist, internist, or in some cases a primary care physician, if evidence can be shown of previous assessment by one of these specialists. TOH only assesses adult patients (≥16 years of age). Referrals for patients with post-concussion syndrome to either the MDC or RAD clinics are redirected to two specialized concussion and traumatic brain injury (TBI) clinics, which are both based out of TOH.

Once accepted by the MDC, patients are mailed a set of questionnaires with notification of their appointment. In addition, instructions are sent informing them of the format of their upcoming assessment along with how to complete the various patient-centred questionnaires. The completed questionnaires are collected by the team on the day of the MDC appointment and the scores contribute to the MDC medical record. As standard, patients are requested to complete the Patient Health Questionnaire-9 (PHQ-9), Generalized Anxiety Disorder-7 (GAD-7), and Dizziness Handicap Inventory (DHI)]. The PHQ-9 is a validated, self-reported tool to evaluate for depressive symptoms [9]. The GAD-7 is a validated, self-reported tool to assess for symptoms of generalized anxiety disorder [10]. The DHI is a validated 25-item self-reported questionnaire that evaluates the physical, emotional, and functional impact of dizziness symptoms on daily life [11].

In the MDC, patients are first assessed by the nurse and a resident or fellow; this assessment is followed by discussion of the case with all members of the allied healthcare team. The team then performs a final assessment as a group, and through consensus, arrives at a final diagnosis and, in conjunction with patient input, a treatment plan is developed. This plan communicated to the patient using various resources, including a treatment roadmap and follow- up form. A comprehensive detailed summary letter is shared with the both the patient’s primary care physician and referring physician, along with any other allied health professionals or other specialists involved in the patient’s care. If any further investigations are deemed necessary, they are ordered and the results followed up with by an MDC team member or the referring physician as appropriate. Routinely, follow-up care is delegated to the referring physician unless further investigations or treatment require specific expertise. In general, the total assessment time for each patient is 1–1.5 h, with a total of 4–5 patients being seen per weekly clinic.

The RAD clinic is run by the same Staff as the MDC, and accepts referrals from TOH emergency departments exclusively for patients with dizziness of less than 4-weeks duration and no previous history of dizziness. Patients are usually seen within two weeks of referral. Referrals that do not follow the RAD clinic criteria are redirected, as indicated, to neurology or otolaryngology outpatient clinics. The same local network of trained vestibular physiotherapists supports the RAD clinic, providing early access to vestibular rehabilitation or canalith repositioning therapy as needed.

Each patient at the RAD clinic has a full medication history, vitals, and postural vitals collected by a nurse, followed by a video head impulse test (VHIT) performed by a vestibular technician. Similar to the MDC, they are then assessed first by a resident/fellow, discussed by the allied health team, and reassessed as needed. Through consensus a diagnosis is made and with input from the patient, a management plan formed. Patients typically follow up with their primary care physician, unless specialized follow up is felt appropriate. For example, patients thought to have suffered a first attack of Meniere’s disease might have follow up arranged with otolaryngology, including audiological assessment. Likewise, patients with suspected vestibular migraine may have follow up with neurology. Similar to the MDC, each patient assessment typically takes 1–1.5 h, including the VHIT and the RAD clinic sees approximately 6–8 patients per week.

## Methods & Analysis

We performed a retrospective review of medical charts from all patients presenting to the MDC and RAD clinic at TOH from July 1, 2015 to August 1, 2017. For both clinics, we extracted demographic variables from the electronic patient database, including patient age and gender. For the MDC, we extracted additional clinic-specific variables, including scores on the PHQ-9, GAD-7, and DHI. A final diagnosis for each patient was developed following synthesis of the original clinical assessment, any further testing that was deemed necessary, and subsequent clinical assessments documented in the medical record.

For the RAD clinic, we extracted clinic-specific variables, including duration of dizziness and referral diagnoses. We extracted diagnostic test results from emergency department visits, including results of the HINTS (Head-Impulse—Nystagmus—Test of Skew) exam battery and the Dix-Hallpike test. In addition, we extracted CT and MRI results and also reported the final RAD clinic diagnosis. Again, final diagnosis was reached following integration of the clinical assessment, VHIT, and any contributory investigations performed by the referring physician. With the RAD clinic cohort, we compared demographic and diagnostic data, and the accuracy of referral diagnoses.

Collected data were analysed using descriptive statistics. Categorical variables were analysed using frequency histograms, measures of central tendency, and measures of dispersion. Pearson chi-square testing was employed to compare proportions of patients between clinics. In addition, non-parametric statistical analyses (i.e. Mann-Whitney U) were used to compare variables between clinics. An alpha of < 0.05 was chosen to denote statistical significance. All data collection forms and protocols were approved by the Ottawa Health Science Network Research Ethics Board (20170751-01H).

## Results

### Multidisciplinary dizziness clinic (MDC)

**Fig. 1:**
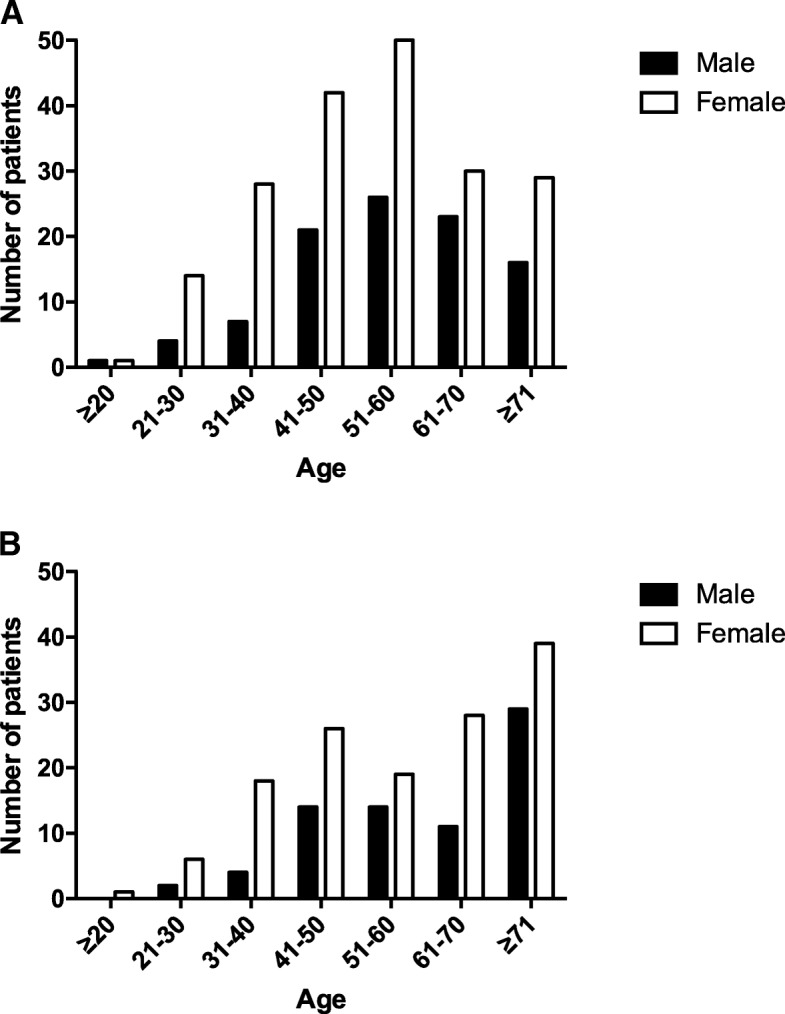
Gender and age distribution of patients seen in the MDC and RAD clinic. **a**: Gender and age distribution in the MDC. **b**: Gender and age distribution in the RAD clinic

Two-hundred and ninety-two (*n* = 292) patients were seen in the MDC. Of the patients seen in the MDC, 66.4% (*n* = 194) were female and 33.6% (*n* = 98) were male. The median age of patients seen in the MDC was 55 years old (range: 17–90 years old). Patients in the MDC were predominantly 51–60 years old (Fig. 1, Graph A).

**Fig. 2:**
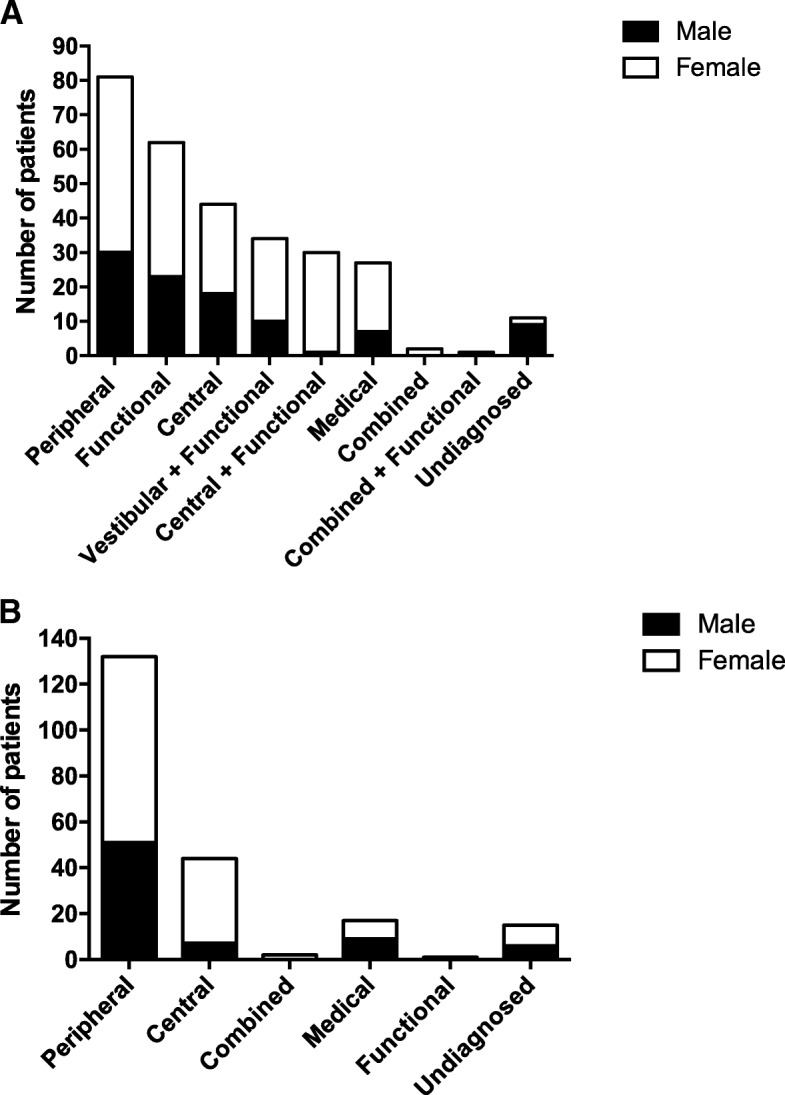
Distribution of diagnoses in the MDC and RAD clinic. **a**: Distribution of diagnoses in the MDC. **b**: Distribution of diagnoses in the RAD clinic

We found that 27.7% (81/292) of patients in the MDC were categorized as having vestibular dizziness, which encompassed benign paroxysmal positional vertigo (BPPV), which was diagnosed by history and/or positive positional testing; poor and incomplete dynamic compensation from previous acute vestibular neuritis, which was diagnosed through combination of clinical assessment and videonystagmography (VNG), rotary chair, and VHIT; Meniere’s disease, which encompassed both probable and definite types; and bilateral vestibular hypofunction (BVH), which was diagnosed through clinical exam and use of VNG, rotary chair, and VHIT. We found that 21.2% (62/292) of patients were categorized as having symptoms solely as a result of functional dizziness with no identifiable inciting vestibular or central event. These patients primarily suffered from persistent postural-perceptual dizziness (3PD) or stress/anxiety-related dizziness not meeting criteria for 3PD, which was diagnosed based on clinical assessment and negative diagnostic testing. We found that 15.1% (44/292) of patients experienced central dizziness, which mainly included migraine, vestibular migraine and chronic migraine, but also a small number of patients with rarer disorders such as multiple sclerosis, spinocerebellar ataxia syndromes, or cerebellar ataxia neuropathy vestibular areflexia syndrome (CANVAS). In addition, 9.3% (27/292) of MDC patients diagnosed with medical dizziness had multifactorial medical-related dizziness, with orthostasis (diagnosed using postural vitals performed in clinic) and diabetic neuropathies (diagnosed through clinical neurologic examination and/or previously diagnosed by other physicians) being large contributors. There were 11 patients (3.8%) where no definitive diagnosis was reached. The rest of the patients (67/292, 23.0%) had multiple combined diagnoses, most often 3PD, with an identifiable or diagnosed inciting vestibular or central disorder, either persistent or previously resolved (Fig. 2, Graph A). 3PD, either as the main or comorbid diagnosis, affected 44.2% (129/292) of patients in the MDC as a whole.

Eight patients declined to complete the DHI. Stratification of results from the DHI showed that 15.1% (43/284) of all MDC patients had DHI scores from 61 to 70, which is suggestive of a severe disability in these patients with dizziness. Of the patients diagnosed with functional dizziness, 25.6% (32/125) had a DHI from 61 to 70, while only 6.33% (5/79) of patients with vestibular dizziness had a DHI from 61 to 70. The median DHI score of patients was 52. Ten patients declined to complete the PHQ-9. We found that 58.2% (164/282) of patients in the MDC experienced minor or moderate depressive symptoms, while 9.2% (26/282) of patients experienced severe depressive symptoms. Ten patients declined to complete the GAD-7. We found that 40.2% (113/282) of patients had minor anxiety symptoms, while 13.9% of patients (39/282) experienced severe anxiety symptoms. We found that 11.3% (14/124) of patients diagnosed with functional dizziness experienced combined symptoms of severe anxiety and depression. Moreover, we found that patients with severe (mean = 84.5, SD = 4.29) or moderate (mean = 75.1, SD = 2.61) depression had a significantly higher mean DHI than patients with minimal depressive symptoms (mean = 36.1, SD = 14; t (188) = 17.5, *p* < 0.0001; t (192) = 15.2, *p* < 0.0001, respectively). Likewise, patients with severe (mean = 81.7, SD = 4.55) or moderate (mean = 68.8, SD = 3.63) anxiety had a significantly higher DHI than patients who did not experience anxiety symptoms (mean = 31.4, SD = 11.8; t (166) = 26, *p* < 0.0001; t (176) = 21.8, *p* < 0.0001, respectively).

We found that female patients had significantly worse DHI compared to male patients in the MDC (mean = 53.2, SD = 21 vs. mean = 47.1, SD = 21; t (282) = 2.27, *p* = 0.0239). Neither PHQ9 nor GAD7 scores differed significantly between male and female patients in the MDC.

### Rapid access dizziness (RAD) clinic

Two-hundred and eleven patients (*n* = 211) were seen in the RAD clinic between July 012015 and August 312,017. Of the patients seen in the RAD clinic, 64.9% (137/211 patients) were female and 35.1% (74/211 patients) were male. The median age of patients seen in the RAD clinic was 61 years old (range: 18–104 years old). Furthermore, 32.2% of patients (68/211 patients) presenting to the RAD clinic were 71 years or older (Fig. 1, Graph B).

The majority of patients (62.6% or 132/211) in the RAD clinic were categorized as having vestibular disorders. Four patients with a vestibular cause of dizziness had unclear diagnoses and were therefore excluded from descriptive analyses of the vestibular patient cohort. Of the patients with vestibular dizziness, 56.3% (72/128) had BPPV, 42.2% (54/128) had suffered an episode of acute vestibular neuritis or labyrinthitis, and 1.56% (2/128) had had a possible first attack of Meniere’s disease. Central dizziness affected 15.6% (33/211) of patients in the RAD clinic. Of the patients diagnosed with central dizziness, 90.9% (30/33) had vestibular migraine and 9.10% (3/33) had had a transient ischemic attack (TIA). We also found that the final diagnosis made in the RAD clinic was concordant with the initial diagnosis made in the emergency department (ED) in only 25% of patients (*n* = 52). Final diagnosis was reached by the team as a result of clinical history and examination, as well as results of the VHIT, and included information from any investigations that had already been performed (Fig. 2, Graph B).

In patients presenting to the RAD clinic, 44.8% of patients (94/210) had a CT scan completed. Of these patients, 98.9% (93/94) had a normal CT scan, while one patient had an abnormal finding on CT scan (subdural hematoma in a patient with a dizziness induced fall and head trauma). We found that 33.6% (71/211) had HINTS completed in the ED, while 54.5% (115/211) had the Dix-Hallpike manoeuvre completed. Furthermore, 15.6% (33/211) had both the HINTS and the Dix-Hallpike manoeuver completed in the ED, while 55% (116/211) had neither clinical assessment completed.

### Comparison of the MDC and RAD clinics

We demonstrated a statistically significant difference in the distribution of ages between the MDC and RAD clinics (*χ*^2^ = 23.6, *p* = 0.0006) with the RAD clinic (median age = 61) having a significantly older cohort of patients when compared to the MDC (median age = 55; U = 24,999, *p* = 0.0003). We also found that both the MDC and RAD clinic had a similar proportion of male and female patients (*χ*^2^ = 0.124, *p* = 0.725) with a ~ 2:1 female-to-male ratio in both clinic cohorts. We found a significant difference in the distribution of diagnoses between the MDC and RAD clinic (*χ*^2^ = 71.1, *p* < 0.0001). Our findings demonstrate that peripheral vestibular dizziness was more commonly diagnosed in the RAD clinic, while functional dizziness was more commonly diagnosed in the MDC.

## Discussion

We described the demographic and clinical characteristics of patients who presented to two tertiary dizziness clinics at TOH in Ottawa, Ontario, Canada. The MDC, which started in July 2015, serves specialist-referred chronically dizzy patients via a multidisciplinary team of medical specialists, nurses, physiotherapists, and audiologists. In contrast, the RAD clinic, which started in September 2016, serves acutely dizzy patient populations who have been referred by an emergency physician.

A comparison of the demographic characteristics of patients demonstrates that the majority of patients were female in both the MDC and RAD clinic. These findings are consistent with previous studies citing an increased lifetime prevalence of moderate or severe dizziness or vertigo in females [12]. In addition, females with dizziness and unsteadiness have been reported to experience a higher degree of disability and psychiatric co-morbidity when compared to males [13]. Our results demonstrated that females in the MDC had worsened dizziness symptoms when compared to males, but this difference did not extend to indices of depression and anxiety. The majority of patients seen in the MDC were 51–60 years old. Studies of other MDC for patients with dizziness demonstrate a similar pattern of demographic characteristics, including a predominance of female patients and patients aged 50 years and older [7, 14–16]. In the RAD clinic, we found that approximately one-third of patients were 71 years or older, which is consistent with the typical age of acutely dizzy patients in an emergency setting [17, 18].

We also reported on clinical variables, including the distribution of diagnoses and types of investigations, of patients presenting to the MDC and RAD clinics. In the MDC, we found that 28% of patients suffered from peripheral dizziness, including BPPV, vestibular neuritis, Meniere’s disease, and BVH. In addition, 21% of patients had functional dizziness, of which, the majority patients were diagnosed with 3PD. These findings are consistent with previously published studies of MDCs, which have noted high rates of peripheral dizziness, especially BPPV, and a slightly lower frequency of central dizziness [7, 14–16]. Moreover, our results highlight the impact of comorbid psychiatric disorders, including severe anxiety and depression, on chronically dizzy patients seen in the MDC. Similarly, studies have established a relationship between psychiatric disorders and chronic dizziness [19–21]. We employed the DHI to demonstrate that many patients with chronic dizziness reported severe disability. An association has previously been reported between chronic dizziness, the severity of dizziness, the severity of comorbid anxiety and depression, and quality of life [22].

Due to the referral process of the MDC, the patient population is likely biased towards a more homogeneous subset of chronically dizzy patients. This does provide a unique opportunity to study this patient population in detail. This preliminary analysis has shown that 23% of patients present with 3PD symptoms with an identifiable inciting vestibular or central disorder. In fact, almost half of the patients in the clinic have 3PD either as a main or comorbid diagnosis. Most commonly, this triggering disorder is vestibular neuritis (vestibular) and migraines (central). Unfortunately, data is not available on how many of these inciting triggers resolved or persisted. Anecdotally however, the senior author would note that in a majority of cases, vestibular triggers had resolved, while migraines were persistent, and often worsened, by the development of 3PD.

In the RAD clinic, we reported that the majority of patients suffered from peripheral dizziness, of which, over 50% suffered from BPPV and over 40% had vestibular neuritis or labyrinthitis. We also reported that the next largest subset of RAD clinic patients was diagnosed with central dizziness, mostly vestibular migraine (90,9%). Our reported diagnostic frequencies are consistent with studies of acutely dizzy patients, which have identified a high rate of BPPV in the emergency setting [17, 23]. We also found that only 25% of diagnoses made in the RAD clinic were consistent with those made in the emergency department, a discrepancy that has been noted in other studies [24, 25]. This result suggests that improvements need to be made in the diagnosing and managing dizzy patients in an acute setting. Moreover, we demonstrate that CT scans performed in acutely dizzy patients did not yield informative diagnostic information. Previous findings have suggested that routine CT scans for patients presenting with dizziness provide little diagnostic value [26] and may not be cost effective [27]. We also report that approximately two-thirds of patients did not have HINTS completed, even though HINTS has been demonstrated to be more sensitive than MRI for diagnosing acute vestibular syndrome [28]. This finding is consistent with a recent single-centre retrospective review demonstrating an under-utilization of HINTS in the ED for peripheral vertigo [29]. At our hospital, further quality improvement studies are being undertaken to address both use of CT scanning and the use of HINTS examination in acute dizziness.

## Conclusion

In a tertiary care centre in Canada, the majority of patients referred to both acute and chronic dizziness clinics were women over 50 years of age. In chronic dizziness populations, functional comorbidity is common, likely highlighting the need for concurrent psychiatric/psychologic care. In acute dizziness peripheral causes form the majority, while clinical examinations remain underused and diagnostic imaging remains overused, with most cases lacking concordance between emergency department and specialist diagnoses. This suggests further room for improvement in the assessment and management of patients with dizziness in the emergency department. Both acute and chronic dizziness clinics can provide unique and effective approaches to diagnosing and managing patients with dizziness of various aetiologies.

## Abbreviations

BPPV: Benign paroxysmal positional vertigo
BVH: Bilateral vestibular hypofunction
CANVAS: Cerebellar ataxia neuropathy vestibular areflexia
DHI: Disability Handicap Index
GAD-7: Generalized Anxiety Disorder-7
HiNTS: Head-Impulse—Nystagmus—Test of Skew
MDC: Multidisciplinary clinic
PHQ-9: Patient Health Questionnaire-9
PPPD: Persistent postural-perceptual dizziness
RAD: Rapid access dizziness
TIA: Transient Ischemic Attack
TOH: The Ottawa Hospital
VHIT: Video Head Impulse Testing
